# Cluster analysis unveils a severe persistent respiratory impairment phenotype 3-months after severe COVID-19

**DOI:** 10.1186/s12931-022-02111-9

**Published:** 2022-08-02

**Authors:** Jeanne-Marie Perotin, Fabien Gierski, Lois Bolko, Sandra Dury, Sarah Barrière, Claire Launois, Maxime Dewolf, Stéphane Chouabe, Eric Bongrain, Davy Picard, Eric Tran, Yoann N’Guyen, Bruno Mourvillier, Amélie Servettaz, Amandine Rapin, Claude Marcus, François Lebargy, Arthur Kaladjian, Jean-Hugues Salmon, Gaetan Deslee

**Affiliations:** 1 Department of Pulmonary Medicine, University Hospital of Reims, Reims, France; 2 INSERM P3Cell UMR-S1250, SFR CAP-SANTE, University of Reims Champagne Ardenne, Reims, France; 3 Department of Psychiatry, Marne Public Mental Health Institution & Reims University Hospital, Reims, France; 4 Cognition Health Society Laboratory (C2S – EA 6291), SFR CAP-SANTE, University of Reims Champagne Ardenne, Reims, France; 5 Faculty of Medicine, Rheumatology Department, University of Reims Champagne-Ardenne, Maison Blanche Hospital, Reims University Hospitals, 3797 Reims, EA France; 6 EA7509 IRMAIC, University of Reims Champagne-Ardenne, Reims, France; 7 Department of Pulmonary Medicine, Charleville Mézière Hospital, Charleville Mézière, France; 8 Department of Pulmonary Medicine, Epernay Hospital, Epernay, France; 9 Department of Pulmonary Medicine, Chalons en Champagne Hospital, Chalons en Champagne, France; 10 Department of Infectious and Tropical Diseases, Reims University Hospital, Reims, France; 11 Medical Intensive Care Unit, University Hospital of Reims, Reims, France; 12 Internal Medicine, Clinical Immunology and Infectious Diseases Department, University Hospital Centre, Reims, France; 13 Physical and Rehabilitation Medicine Department, Sebastopol Hospital, University Hospital of Reims, Reims, France; 14 Faculty of Medicine, University of Reims Champagne Ardennes, 3797 VieFra, Reims, UR France; 15 Department of Radiology, University Hospital of Reims, Reims, France

**Keywords:** COVID-19, D_LCO_, Sarcopenia, Post-traumatic stress disorder, Clustering

## Abstract

**Background:**

The mid-term respiratory sequelae in survivors of severe COVID-19 appear highly heterogeneous. In addition, factors associated with respiratory sequelae are not known. In this monocentric prospective study, we performed a multidisciplinary assessment for respiratory and muscular impairment and psychological distress 3 months after severe COVID-19. We analysed factors associated with severe persistent respiratory impairment, amongst demographic, COVID-19 severity, and 3-month assessment.

**Methods:**

Patients with severe SARS-CoV-2 pneumonia requiring ≥ 4L/min were included for a systematic 3-month visit, including respiratory assessment (symptoms, lung function, CT scan), muscular evaluation (body composition, physical function and activity, disability), psychopathological evaluation (anxiety, depression, post-traumatic stress disorder-PTSD) and quality of life. A cluster analysis was performed to identify subgroups of patients based on objective functional measurements: D_LCO_, total lung capacity and 6-min walking distance (6MWD).

**Results:**

Sixty-two patients were analysed, 39% had dyspnea on exercise (mMRC ≥ 2), 72% had D_LCO_ < 80%, 90% had CT-scan abnormalities; 40% had sarcopenia/pre-sarcopenia and 31% had symptoms of PTSD. Cluster analysis identified a group of patients (n = 18, 30.5%) with a severe persistent (SP) respiratory impairment (D_LCO_ 48 ± 12%, 6MWD 299 ± 141 m). This SP cluster was characterized by older age, severe respiratory symptoms, but also sarcopenia/pre-sarcopenia, symptoms of PTSD and markedly impaired quality of life. It was not associated with initial COVID-19 severity or management.

**Conclusions and clinical implication:**

We identified a phenotype of patients with severe persistent respiratory and muscular impairment and psychological distress 3 months after severe COVID-19. Our results highlight the need for multidisciplinary assessment and management after severe SARS-CoV-2 pneumonia.

*Trial registration* The study was registered on ClinicalTrials.gov (May 6, 2020): NCT04376840

**Supplementary Information:**

The online version contains supplementary material available at 10.1186/s12931-022-02111-9.

## Background

Almost 2 years after the onset of SARS-CoV-2 pandemic, identification of patients at risk of mid and long-term sequelae remains an unmet need. The first wave of SARS-CoV-2 infections hit the North-East of France in March 2020 and had a major impact in terms of hospitalizations and mortality. The severity of general and respiratory symptoms, the worrying radiological findings, the length of hospitalization, and the massive weight loss raised fears of COVID-19-induced long-term respiratory, muscular and psychologic sequelae.

Studies performed after other coronaviruses epidemics (SARS-CoV and MERS-CoV) identified mid and long-term impact on respiratory function, including altered diffusion lung capacity for carbon monoxide (DLCO) [1], and persistent radiological alterations 3 [1, 2] and 12 months after infection [3]. A major psychiatric impact has been identified in patients after SARS-CoV: 50% had anxiety, 20% had depression and/or sleep disturbance; post-traumatic stress syndrome was described [4, 5].

Little is known about mid and long-term respiratory and psychological sequelae in survivors of severe COVID-19 [6]. Recent studies including survivors of both severe and non-severe forms of COVID-19 identified impaired lung function 3–4 months after COVID-19, including a decrease in forced vital capacity (FVC), D_LCO_, total lung capacity (TLC), 6-min walking distance (6MWD), and impairment in respiratory muscle strength. These alterations were associated with the presence of pneumonia [7], the severity of COVID-19, the length of hospital stay [8], and the severity of CT scan score on admission [8]. Other studies observed similar dyspnea scores and pulmonary functions in intensive care unit (ICU) and non-ICU patients, although ICU patients had a higher prevalence of abnormal CT scan 3 months after discharge [9]. Therefore, mid-term clinical and functional sequelae of severe COVID-19 appear highly heterogeneous [10], and factors associated with mid-term sequelae in this population are not known.

In this monocentric prospective study, we used a multidisciplinary approach to assess both respiratory and psychological impairment 3 months after severe SARS-CoV-2 pneumonia. Given the observed impact of severe COVID-19 on weight loss and muscular weakness, we also analysed sarcopenia and performed functional assessments. Finally, we analysed factors associated with persistent respiratory impairment to identify a phenotype of “high-risk” patients to monitor closely.

## Methods

### Patients

We prospectively included adult patients with severe SARS-CoV-2 pneumonia requiring hospitalization. Inclusion criteria were (1) confirmed diagnosis of SARS-CoV-2 infection via reverse transcription-polymerase chain reaction testing or suggestive computed tomography results with no alternative diagnosis and (2) patients with marked hypoxemia requiring oxygen therapy ≥ 4L/min to maintain oxygen saturation above 90%, high flow nasal oxygen or mechanical ventilation. Exclusion criteria included the inability to complete questionnaires or to perform lung function tests. Information was provided and patients were included for a systematic 3-month multidisciplinary assessment visit. Written informed consent was obtained for each patient. This study was approved by the French ethics committee (Comité de Protection des Personnes du Nord-Ouest IV, number 2020-A01260-39, 30 April 2020; NCT04376840).

Demographic data and medical history were recorded, as well as initial clinical, biological, and radiological characteristics of SARS-CoV-2 pneumonia and its therapeutic management.

### 3-month assessment

The respiratory assessment included respiratory symptoms, lung function, and CT scan. Briefly, dyspnea was assessed by the modified Medical Research Council (mMRC) breathlessness scale questionnaire, cough and sputum were assessed by the Cough And Sputum Assessment Questionnaire (CASA-Q). Pulmonary function tests were performed including TLC, FVC, FEV_1_, D_LCO_, 6MWD, and arterial blood gases at rest. A composite physiologic index (CPI) was calculated as previously described (CPI = 91 – (– 0.65 × D_LCO_ %pred) – (0.53 × FVC %pred) + (0.35 × FEV_1_%pred)) [11].

A Thoracic CT scan was performed for all patients. The reconstructed 1 mm thick images were analysed by two independent investigators (JMP, SD) with a third investigator (GD) in case of disagreement. Final scores were established by consensus. Interstitial pneumonia was scored for each lung segment as previously described. The severity was classified into four grades (grade 0: no involvement; grade 1: 50% or less of the lung segment involved by residual ground-glass opacification with/without reticulation; grade 2: more than 50% lung segments involved by ground-glass opacification with/without reticulation; grade 3: evidence of lung fibrosis such as thick parenchymal bands, architectural distortion, traction bronchiectasis). In the statistical analysis, individual segmental scores were added together to give a summation score (maximum 54 points) [12].

The muscular evaluation included body composition measures, physical function and activity, and disability assessment. Briefly, total and regional lean, fat, and bone masses were evaluated using a whole-body Dual Energy X-absorptiometry scanner (Hologic, Horizon A). Appendicular lean mass index (ALMI) was used as a surrogate measure of total body muscle mass. Grip strength was measured using a hand dynamometer (JAMAR®) with a standardized position. SARC-F questionnaire was used as a sarcopenia screening tool [13]. Pre-sarcopenia was defined by a decrease in the ALMI or grip strength only [14]. Sarcopenia was defined by a decrease in the ALMI < 7 kg/m^2^ in men and < 5.5 kg/m^2^ in women and a decrease of grip strength < 27 kg in men and 18 kg in women according to the European Working Group on Sarcopenia in Older people (EWGSOP) [13]. Ricci and Gagnon test (RG; Montreal University, modified by Laureyns and Séné) was used for the screening of physical activity in daily life. RG score > 18 identifies active patients. Fatigue was screened by FACI-T questionnaire [15].

The psychopathological evaluations were performed using questionnaires assessing anxiety and depression (HAD) and post-traumatic stress disorder (PCLS). Scores ≥ 8 for depression (HAD-D) or anxiety (HAD-A), and ≥ 44 for PCLS were considered abnormal [16, 17]. Missing values were imputed by using the linear trend at point method (with a limit of 20% of missing values). Reliability were as follows: HAD-D, α = 0.86; HAD-A, α = 0.77; PCLS, α = 0.92.

Health-related quality of life was assessed using the 12-item Short-Form Health Survey (SF-12) and the Saint George’s Respiratory Questionnaire (SGRQ).

### Statistical analysis

The primary objective was to describe respiratory, psychological, and muscular impairment 3 months after severe SARS-CoV-2 pneumonia. Secondary objectives were to identify factors associated with severe persistent respiratory impairment using cluster analysis, amongst demographic, initial COVID-19 severity, and 3-month assessment characteristics.

Data are expressed as mean ± standard deviation, median [interquartile range] depending on distribution, or number and percentages. Associations were analysed using the Student t-test, Wilcoxon test, Chi2 test, exact Fisher test, Pearson test, or Spearman test depending on the data nature and distribution. Analysis was performed using SPSS Statistics v27. p-value < 0.05 was considered significant.

A cluster analysis was performed to identify subgroups of patients according to clinical variables. We used the two-step clustering function provided by IBM-SPSS (version 27). This classification method automatically identified subgroups of patients using three variables depending on objective measurements of lung function: D_LCO_ (% of predicted value), TLC (% of predicted value), and exercise capacity (6MWD, meters). At the first step, the log-likelihood distance was used to assign participants to the cluster leading to the largest log-likelihood. At the second step, the Bayesian Information Criterion (BIC) was used to assess multiple cluster solutions and automatically determine the optimum number of clusters. As clustering is sensitive to outliers and multicollinearity issues, we computed Mahalanobis distance for the D_LCO_, TLC, and 6MWD scores as well as variance inflation factors (VIFs). None of the participants were under the critical χ^2^ p-value (p < 0.001) on Mahalanobis distance; while VIFs were below the standard cut-off (≥ 2.5; range 1.22–1.69; [18]) indicating that there was no multicollinearity issue. Two patients did not perform D_LCO_ measurements and 1 patient did not perform 6MWD measurement and therefore were not included in this analysis.

## Results

### Patients

A total of 65 patients were included. One patient was excluded (no severe pneumonia) and 2 patients did not perform a CT scan at 3 months. Sixty-two patients were analysed.

Patients were mainly men (63%), with a mean age of 63 ± 12 years. Comorbidities were frequent including cardiovascular disease (60%), hypertension (52%), obesity (34%), and diabetes mellitus (24%). The presence of previous respiratory comorbidities was uncommon (Table 1).

**Table 1:** Patient’s demographic characteristics and initial COVID-19 severity data

Characteristic	
Number of patients	62
Male	41 (63.1)
Age, years	63.0 ± 12.4
BMI, kg/m^2^	29.3 ± 5.7
Smoking history	
Current or ex-smoker	30 (48.4)
Pack-years	36.3 ± 35.0
Comorbidities	
Obesity	21 (33.9)
Diabetes mellitus	15 (24.2)
Cardiovascular disease	37 (59.7)
Arterial hypertension	32 (51.6)
Immunodepression	4 (6.5)
Respiratory chronic disease	
COPD	4 (6.5)
Asthma	8 (12.9)
Interstitial lung disease	1 (1.6)
Inhaled corticosteroids	8 (12.9)
Covid-19	
Total length for hospital stay, days	19.5 [37.0]
ICU, n	37 (59.7)
ICU stay length, days	13.0 [13.5]
High-flow nasal oxygen, n	34 (54.8)
High-flow nasal oxygen, days	4.5 [6.0]
Intubation, n	19 (30.6)
Intubation, days	13.0 [20.0]
Physical medicine and rehabilitation unit, n	23 (37.1)
Complications	
ARDS	18 (29.0)
Pulmonary embolism	9 (14.5)
Ventilator-associated pneumonia	9 (14.5)
Acute cardiac insufficiency	3 (4.8)
Pneumothorax	2 (3.2)

Data are expressed as numbers (percentages) or median [interquartile range]

BMI: body mass index; COPD: Chronic Obstructive Pulmonary Disease; ICU: Intensive Care Unit; ARDS: Acute Respiratory Distress Syndrome

In March 2020 (corresponding to the local epidemic peak), no clear recommendation was available regarding drug treatment. All patients were treated with antibiotics and antithrombotic drugs, all but 2 received antiviral drugs, and 81% received systemic corticosteroids. All patients required oxygen supplementation ≥ 4L/min, 60% required hospitalization in ICU, 55% received high flow nasal oxygen therapy. Intubation and invasive mechanical ventilation were necessary for 19 patients (31%). The median hospitalization length was 19.5 days.

### 3-month respiratory and muscular impairment and psychological distress

Twenty-four (38.7%) patients described significant persistent dyspnea in daily living (mMRC ≥ 2), 45 (72%) had D_LCO_ < 80%, 16 (26%) had D_LCO_ < 60% (Table 2). Sarcopenia or pre-sarcopenia was identified in 25 patients (40.3%). The HAD questionnaire revealed significant symptoms of anxiety and depression among respectively 14.5% and 21% of the patients, 19 patients (30.6%) exhibited scores above the standard cut-off on the PCLS, suggesting potential post-traumatic-stress disorders (Table 2).

**Table 2:** Respiratory, muscular, and psychological characteristics 3 months after severe Covid-19

Characteristic	
n	62
Respiratory assessment	
Dyspnea (mMRC score ≥ 2)	24 (38.7)
FEV_1_, % pred	94.8 ± 19.0
FVC, % pred	95.6 ± 20.3
TLC, % pred	99.7 ± 18.9
DLCO, % pred	67.7 ± 17.1
< 80%	45 (72.6)
< 60%	16 (25.8)
6MWD, m	425 ± 127
6MWD < 350 m	13 (21.0)
CT score	7 [13]
Sarcopenia assessment	
SARC-F	1 [2.5]
Sarcopenia or presarcopenia	25 (40.3)
Psychopathological assessment	
HAD Anxiety score ≥ 8	9 (14.5)
HAD Depression score ≥ 8	13 (21.0)
PCLS score ≥ 44	19 (30.6)

Data are expressed as numbers (percentages), mean ± SD, or median [interquartile range]

HAD: Hospital Anxiety and Depression scale; PCLS: Post-traumatic stress disorder CheckList Scale

**Fig. 1:**
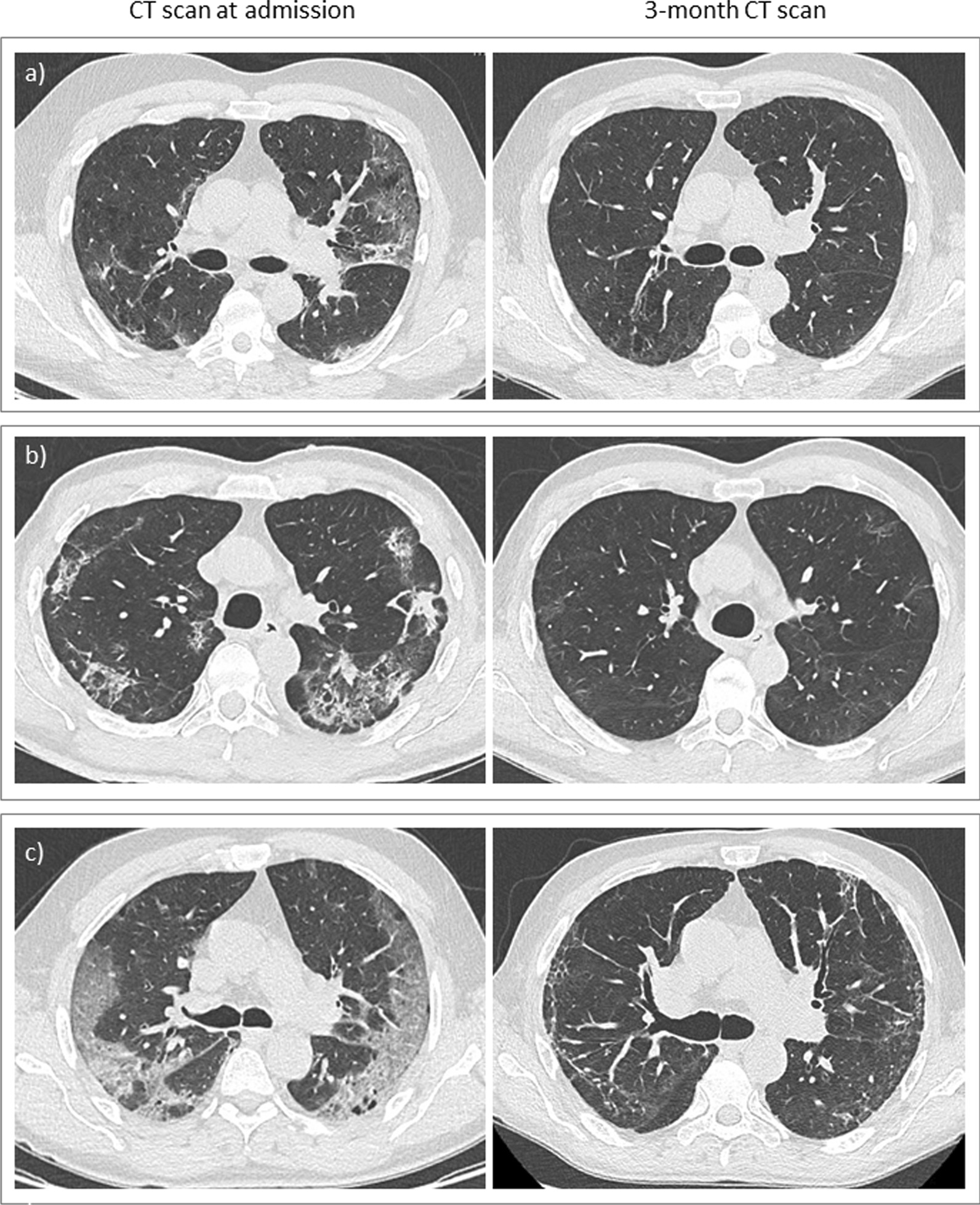
CT-scan features at diagnosis and 3-month assessment in patients with decreased CT-score (**a**, **b**) and patients with increased CT-score (**c**) at 3 months

CT scan was performed with a mean of 118 ± 23 days after the onset of COVID-19 symptoms. Persistent abnormalities were identified in 90.3% of the patients, including ground glass and/or reticulations (38.7%), and fibrosis features (53.2%) characterized by thick parenchymal bands, architectural distortion, and/or traction bronchiectasis (Table 3, Fig. 1). CT scan was normal in 6 patients. When compared with the CT scan performed at admission (n = 47), the 3-month CT scan score improved in 42 cases (89.3%). The 3-month CT-score was not associated with CT-score at diagnosis (p = 0.181) (Table 3).

**Table 3:** CT-scan characteristics at admission and 3 months after COVID-19

		At admission	3-month assessment	p-value
n		47	62	
Delay after the onset of symptoms		12.7 ± 8.0	117.8 ± 23.0	
Radiological findings				
No abnormalities		0 (0.0)	6 (9.7)	0.003
Ground glass and/or reticulations		32 (68.1)	24 (38.7)	
Fibrosis		15 (31.9)	33 (53.2)	
CT-Score				
Total		23 [14]	7 [13]	< 0.0001
Upper right lobe		2.3 [1.8]	1.0 [3.0]	0.056
Middle lobe		2.0 [2.0]	1.0 [1.0]	< 0.0001
Lower right lobe		2.8 [2.0]	2.0 [5.0]	0.949
Upper left lobe		7.0 [6.0]	1.0 [2.3]	< 0.0001
Lingula		3.0 [2.0]	0.8 [1.0]	< 0.0001
Lower left lobe		14.0 [9.5]	1.0 [3.5]	< 0.0001

Data are expressed as numbers (percentages), mean ± SD, or median [interquartile range]

p-values were calculated only for patients with CT-scan both at admission and 3-months assessment (n = 47)

The severity of persistent CT scan abnormalities, as defined by the 3-month CT-score, was associated with a lower D_LCO_ (p 0.02, r^2^ − 0.29) and exercise-induced oxygen desaturation (a drop of 4% or more in SpO2 or SpO2 < 90% during 6 MW test [19]; p = 0.011). CT-score tended to be associated with 6MWD (p = 0.089, r^2^ − 0.22) and CPI (p = 0.082, r^2^ − 0.25). CT-score was not associated with respiratory symptoms, FEV_1_, TLC, or PaO2 at rest (Additional file 1: Table S1).

### Severe persistent respiratory impairment clustering

To determine factors associated with the most severe persistent respiratory impairment at 3 months, we next performed a 2-step clustering of patients depending on objective measurements of lung function and exercise capacity: TLC, D_LCO_, and 6MWD. This analysis resulted in 2 clusters: a “severe persistent respiratory impairment” cluster (SP, n = 18, 30.5%) and a “non severe persistent respiratory impairment” cluster (NSP, n = 41, 69.5%).

**Fig. 2:**
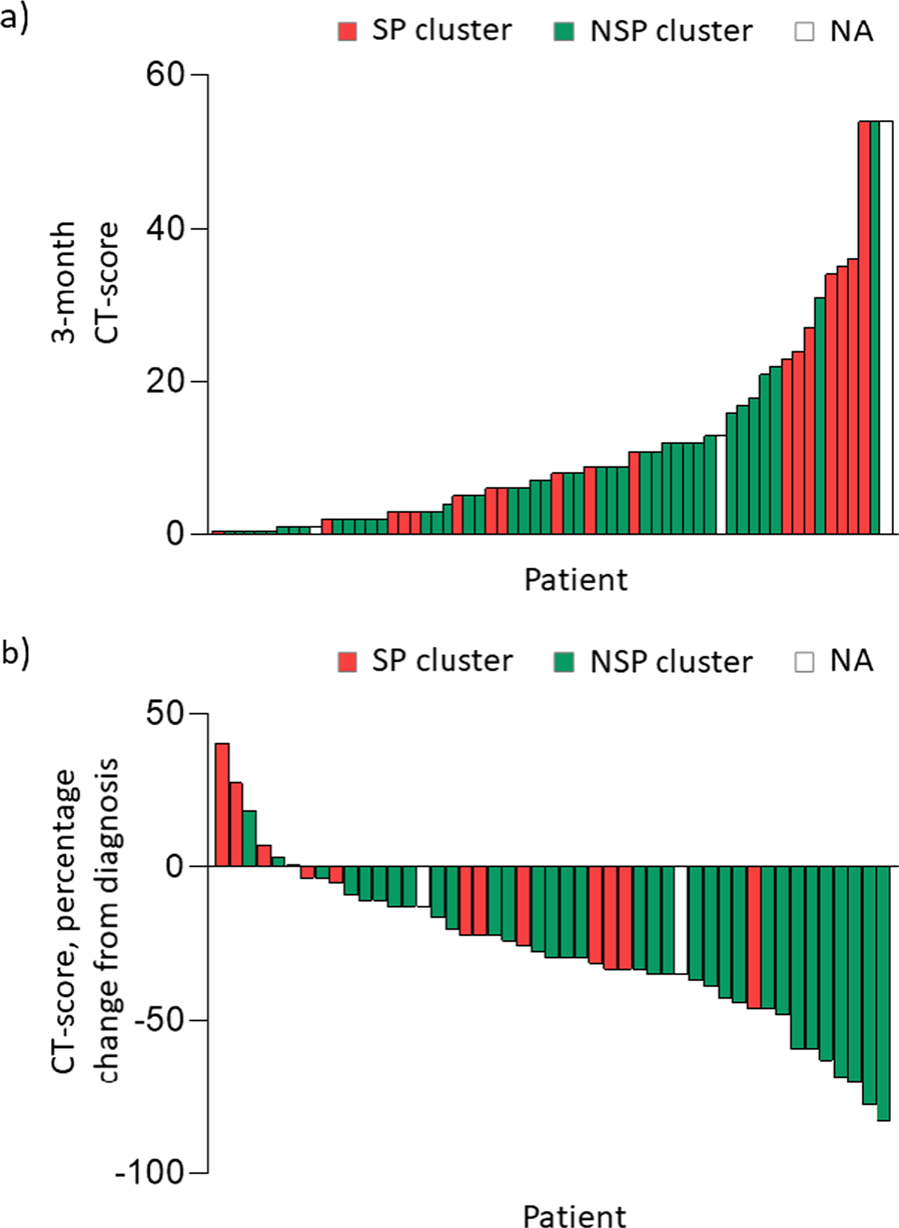
3-month CT-score (**a**) and CT-score change from admission (**b**) in patients in the severe persistent respiratory impairment (SP) cluster (red bars) and the non severe persistent respiratory impairment (NSP) cluster (green bars). Three patients were not included in the clustering analysis: D_LCO_ was not available for 2 patients, 6MWD was not available for one patient (white bars; NA: not applicable)

We first analysed patients’ respiratory phenotypes for each cluster (Table 4). Compared with the NSP cluster, patients in the SP cluster had more severe dyspnea in daily living and more frequent and severe cough. As expected, lung function (FEV_1_, FVC, TLC, D_LCO_) and CPI were significantly impaired as well as the 6MWD in the SP cluster. Compared to the NSP cluster, patients in the SP cluster had a lower PaO2 at rest and higher mean CT-score. Of notes, patients with the highest CT-scores at 3 months were mainly, but not exclusively, in the SP cluster (Fig. 2). The SP cluster also exhibited higher blood inflammation markers a 3 months and impaired quality of life (Table 4, Additional file 1: Table S2).

**Table 4:** Factors associated with the severe persistent (SP) respiratory impairment cluster

		SP cluster	NSP cluster	p-value
n		18	41	
Clustering factors				
DLCO, % pred		47.9 ± 12.2	76.5 ± 10.6	< 0.0001
TLC, % pred		84.0 ± 13.5	106.2 ± 17.7	< 0.0001
6MWD, m		299 ± 141	480 ± 65	< 0.0001
Associated factors				
Demography				
Age		67.8 ± 10.9	60.2 ± 12.5	0.024
COVID-19 features at admission				
Delay from symptoms onset to admission, days		5 [9]	8 [3]	0.014
Diarrhea at diagnosis		6 (3.3)	27 (65.9)	0.021
3-months assessment				
Dyspnea (mMRC score ≥ 2)		11 (61.1)	12 (29.3)	0.021
Cough (CASA-Q)				
Symptom		74.1 ± 21.4	84.8 ± 11.9	0.017
Impact		79.5 ± 23.9	92.6 ± 10.1	0.004
Sputum (CASA-Q)				
Impact		89.6 ± 14.5	92.8 ± 14.0	0.037
Lung function				
FEV_1_, % pred		80.7 ± 13.1	101.1 ± 18.2	< 0.0001
FVC, % pred		77.8 ± 15.3	103.7 ± 17.5	< 0.0001
CPI		46.2 ± 11.1	21.0 ± 8.1	< 0.0001
6MWD < 350 m		11 (61.1)	1 (2.4)	< 0.0001
PaO2, mm Hg		84.8 ± 10.8	92.0 ± 14.6	0.043
CT score		8.0 [25.0]	7.0 [11.0]	0.043
Quality of life				
SGRQ Total		38.3 [27.9]	68.8 [20.1]	0.009
Sarcopenia assessment				
SARC-F		3 [2.5]	0 [0–4]	< 0.001
Grip strenght (women)		15 [11.5]	24 [8.8]	0.017
Grip strenght (men)		26 [15.5]	35 [16.0]	0.003
ALMI (men)		7.6 [1.5]	8.3 [1.3]	0.023
Sarcopenia or presarcopenia		12 (66.7)	12 (29.3)	0.010
Psychological assessment				
PCLS score^a^ ≥ 44		5 (33.3)	4 (10.3)	0.042

Data are expressed as numbers (percentages), mean ± SD, or median [interquartile range]

CPI: Composite Physiologic Index; 6MWD: 6-Minute Walking Distance; SGRQ: Saint Georges Respiratory Questionnaire; ALMI: Appendicular lean mass index; PCLS: Posttraumatic stress disorder CheckList Scale

^a^ SP cluster n = 15, NSP cluster n = 39

The SP cluster was associated with more frequent sarcopenia or pre-sarcopenia, a lower median grip test, and decreased ALMI in men. No difference was observed in fat mass percentage, fatigue, or reported physical activity. Regarding psychological distress, patients in the SP cluster had more frequent post-traumatic stress symptoms (33.3%) when compared with the NSP group (10.3%, p = 0.042). Anxiety tended to be more frequent in the SP group (27.8% vs 10.0% in the NSP group, p = 0.084), while depression scores did not significantly differ between groups.

We next compared clusters depending on demographic data and COVID-19 initial features, severity, and management (Table 4, Additional file 1: Table S3). Compared with the NSP cluster, the SP cluster was characterized by older patients and a trend for more frequent diabetes mellitus. The initial clinical presentation did not differ between groups, except for a shorter delay between the first symptoms and admission in the SP group and more frequent diarrhea at diagnosis in the NSP group. The total length for the hospital stay tended to be longer in the SP group, but ICU hospitalization requirement and length as well as high flow nasal oxygen or invasive mechanical ventilation did not differ between groups. Patients in the SP group needed more frequently a post-acute hospitalization in a department of physical medicine and rehabilitation before discharge (p = 0.055).

## Discussion

Using a multidisciplinary approach to assess respiratory, muscular, and psychological sequelae 3 months after severe SARS-CoV-2 pneumonia, we identified that patients frequently suffered from persistent respiratory symptoms, functional alteration, and CT-scan abnormalities. Impaired D_LCO_ has been previously reported after severe COVID-19. In line with our results, D_LCO_ < 80% was identified in 52% of patients requiring hospitalization [6, 20] and 76–82% of patients requiring ICU [20, 21], 1 to 4 months after COVID-19. D_LCO_ < 60% was reported in 15.5% of patients in a population with less severe initial features of COVID-19 [6]. A very recent study analysing lung function 12 months after severe COVID-19 in patients that did not require mechanical ventilation showed improvement in D_LCO_ over time. However, 33% of the patients in this study had persistent impaired D_LCO_ < 80%. No associated factor was identified except female gender and initial CT peak score [10]. In our study, persistent CT-scan abnormalities were very frequent (90%); however, the majority of patients had an improved CT-score at 3 months. In a previous series of 62 patients requiring ICU hospitalization, 70% of patients had abnormal CT-scan at 3 months [21]. Fibrotic patterns were described in 21% of those patients [21]. In a recent review, around 50% of hospitalised survivors of COVID-19 had CT abnormalities 3 months after acute infection, including ground-glass opacity, parenchymal or subpleural bands, reticulation, fibrotic abnormality and air trapping [22]. In our study, we used a CT-scan score validated in the SARS-CoV epidemic [12], allowing a quantification and follow-up of CT-scan changes overtime, demonstrating a dramatic improvement of CT-scan at 3-month compared to admission. Of note, the term of post-COVID-19 lung fibrosis should be reserved for those with clear evidence of traction bronchiectasis, honeycombing or architectural distortion [23]. We cannot exclude in our study that some CT were classified as “fibrosis” because of thick parenchymal bands only which may overestimate the prevalence of fibrosis. A standardisation of post-COVID-19 CT-scan including a sub-classification of CT appearances such as predominantly ground glass, predominantly fibrotic or mixed ground glass and fibrotic [24] may help to better describe post-COVID lung complications. Whether those interstitial lung features including lung fibrosis will persist in the long term is currently not known and remains to be elucidated.

To analyse factors associated with the most severe respiratory abnormalities at 3 months, we performed clustering analysis based on lung function and exercise capacity and identified a cluster of patients, that we named severe persistent respiratory impairment. This cluster was characterized by marked respiratory functional impairment, decreased exercise capacity, and more pronounced CT-scan abnormalities. When analysing clinical features, we identified that this SP cluster was characterized by a clinical phenotype including older age, respiratory symptoms, frequent sarcopenia/pre-sarcopenia and symptoms of post-traumatic stress disorders, and markedly impaired quality of life. This SP cluster was not associated with initial COVID-19 features, except for less frequent diarrhea at diagnosis and a shorter delay from symptom onset to admission. It must be pointed out that all the patients in our study had a severe form of COVID-19.

We found that the patients in the SP cluster were significantly older than those in the NSP cluster. Older age is a recognised risk factor for severe Covid-19 [25]. However, associations between older age and mid-term respiratory impairment are not clear. Previous studies performed 3–4 months after severe forms of Covid-19 found that age was not associated with functional impairment, DLCO decrease [6], or CT score [21], but with tolerance to exercise, a younger age being associated with a perception of reduced tolerance to physical exercise [6]. Digestive symptoms appear common in the acute forms of Covid-19. In a recent study performed on 300 hospitalised patients, up to 83% of patients described diarrhea [26]. In this study, patients with digestive symptoms presented a longer delay from symptom onset to admission, similar to patients of the NSP group in our analysis. The presence of gastrointestinal symptoms was not associated with mortality in a meta-analysis including more than 55.000 patients with Covid-19 [27]. Associations between initial digestive symptoms and respiratory persistent impairment at 3 months were not described in previous studies.

In our study, sarcopenia/pre-sarcopenia was diagnosed in 67% of the SP group and 30% of the NSP group, much more frequent than in the general population of 60–70 years old (5 to 24%) [14, 28]. Sarcopenia is known to be associated with a poorer prognosis in chronic respiratory diseases [29]. In this population, sarcopenia is associated with manual strength [30, 31], which is a predictor of disability, morbidity, and mortality [32]. In our study, the manual strength was significantly impaired in the SP group when compared with the NSP group. The interpretation of those results may be limited by the unknown pre-COVID-19 sarcopenia/pre-sarcopenia status. However, our results are supported by those from a recent study performed in patients hospitalized for COVID-19, showing that a decrease in grip strength was associated with a severe form of infection [33]. Interestingly, we did not find any difference between fat mass and obesity status between the SP and the NSP groups. Furthermore, the differences observed in the body composition were not associated with physical activity, or the need for ICU care or intubation. Our results suggest that systematic strength assessment using the SARC-F questionnaire and grip test at SARS-COV-2 infection diagnosis and during follow-up may be helpful to identify patients at risk of an impaired outcome at mid-term after a severe COVID-19.

The SP cluster was further characterized by higher levels of psychological distress. More specifically, we found that these patients were more prone to display anxiety (28%) and post-traumatic stress symptoms (PTSS, 33%). A recent meta-analysis focusing on severe COVID-19 survivors found a pooled prevalence of PTSS of 16%, 4–16 weeks after COVID-19 diagnosis [34]. A possible PTSS deterioration between 3 and 6 months after COVID-19 has been suggested [35]. Our results suggest that the patients with severe persistent respiratory impairment may benefit from a close monitoring of mental wellness. Our results also suggest that severe persistent respiratory impairment could be a risk factor of psychological distress.

The persistence of this phenotype over time is a crucial yet unanswered question that requires further investigation. Given the design of our study, the patients were not systematically rescheduled for long-term reassessment, which represents a major limitation. Interestingly, two recent studies decribed 1-year follow-up features of severe hospitalized COVID-19 patients, showing frequent persistent fatigue, dyspnea, and quality of life impairment [36] and also DLCO alteration and persistent interstitial lung abnormalities especially in older patients requiring ventilatory support [37]. Our study is also limited by its single-center design, the potential selection bias of patients who consented to a 3-month assessment, the relatively low number of patients, the absence of pre-COVID-19 data regarding respiratory symptoms, pulmonary function, CT scan, sarcopenia and psychological symptoms, and the absence of systematic assessment of pulmonary arterial hypertension by echography. Despite these limitations, we think that the prospective design, the quantification of CT-scan using a validated CT-score and the cluster analysis provide original data in this field.

## Conclusion

In our study, one-third of the patients exhibited severe persistent respiratory impairment 3-months after a severe SARS-CoV-2 pneumonia. These patients were characterized by a clinical phenotype associating impaired lung function and exercise capacity, respiratory symptoms, sarcopenia/pre-sarcopenia, post-traumatic stress disorders, and markedly impaired quality of life, which was not associated with initial COVID-19 features. Our results highlight the need for multidisciplinary assessment and management after the most severe cases of COVID-19. The persistence of respiratory and muscular impairment and psychological distress over time in those patients is currently under investigation.

## Supplementary Information

**Additional file 1:** **Table S1.** Associations between 3-month CT-score and 3-month respiratory assessment results. **Table S2.** 3-month patient’s characteristics in the severe persistent (SP) respiratory impairment cluster and in the non-severe persistent (NSP) respiratory impairment cluster. **Table S3.** Patient’s demographic characteristics and COVID-19 features at admission in the severe persistent (SP) respiratory impairment cluster and in the non-severe persistent (NSP) respiratory impairment cluster.

## Data Availability

The datasets used and/or analysed during the current study are available from the corresponding author on reasonable request.
