# Supplementary material for: Cluster analysis unveils a severe persistent respiratory impairment phenotype 3-months after severe COVID-19
Source: Respir Res. 2022 Aug 2;23:199. doi: 10.1186/s12931-022-02111-9 (PMC9344257; doi:10.1186/s12931-022-02111-9)
Supplement: Supplementary file 1 — Additional file 1: Table S1. Associations between 3-month CT-score and 3-month respiratory assessment results. Table S2. 3-month patient’s characteristics in the severe persistent (SP) respiratory impairment cluster and in the non-severe persistent (NSP) respiratory impairment cluster. Table S3. Patient’s demographic characteristics and COVID-19 features at admission in the severe persistent (SP) respiratory impairment cluster and in the non-severe persistent (NSP) respiratory impairment cluster. [file 12931_2022_2111_MOESM1_ESM.docx]

Table S1: Associations between 3-month CT-score and 3-month respiratory assessment results

|  | | | | | | |
| --- | --- | --- | --- | --- | --- | --- |
|  |  |  | p value | r² | 95%IC | |
| Symptoms | | |  |  |  |  |
|  | Dyspnea (mMRC score≥ 2) | | 0.106 |  |  |  |
|  | CASA-Q | |  |  |  |  |
|  |  | symptom cough | 0.379 | -0.114 | -0.360 | 0.148 |
|  |  | symptom sputum | 0.872 | 0.021 | -0.239 | 0.278 |
|  |  | impact cough | 0.239 | -0.152 | -0.393 | 0.109 |
|  |  | impact sputum | 0.793 | 0.034 | -0.225 | 0.288 |
| Lung function | | |  |  |  |  |
|  | FEV_1_, % pred | | 0.718 | -0.047 | -0.300 | 0.213 |
|  | FVC, % pred | | 0.486 | -0.09 | -0.339 | 0.171 |
|  | TLC, % pred | | 0.750 | -0.041 | -0.295 | 0.218 |
|  | DLCO, % pred | | 0.027 | -0.285 | -0.509 | -0.026 |
|  | CPI | | 0.082 | -0.248 | -0.499 | 0.041 |
| 6-min walking distance, m | | | 0.089 | -0.220 | -0.453 | 0.041 |
| Desaturation* | | | 0.011 |  |  |  |
| Arterial blood gases at rest | | |  |  |  |  |
|  | PaO2 | | 0.949 | 0.008 | -0.253 | 0.269 |

* exercise-induced desaturation: drop of 4% or more in SpO2 or SpO2<90% during 6MWT

Table S2. 3-month patient’s characteristics in the severe persistent (SP) respiratory impairment cluster and in the non-severe persistent (NSP) respiratory impairment cluster.

|  |  |  | SP cluster | NSP cluster | p-value |
| --- | --- | --- | --- | --- | --- |
| n | | | 18 | 41 |  |
| Respiratory assessment | | |  |  |  |
|  | Dyspnea (mMRC score≥ 2) | | 11 (61.1) | 12 (29.3) | 0.021 |
|  | Cough (CASA-Q) | |  |  |  |
|  |  | Symptom | 74.1 ± 21.4 | 84.8 ± 11.9 | 0.017 |
|  |  | Impact | 79.5 ± 23.9 | 92.6 ± 10.1 | 0.004 |
|  | Sputum (CASA-Q) | |  |  |  |
|  |  | Symptom | 81.9 ± 22.5 | 85.4 ± 18.9 | 0.546 |
|  |  | Impact | 89.6 ± 14.5 | 92.8 ± 14.0 | 0.037 |
|  | Lung function | |  |  |  |
|  |  | FEV_1_, % pred | 80.7 ± 13.1 | 101.1 ± 18.2 | <0.0001 |
|  |  | FVC, % pred | 77.8 ± 15.3 | 103.7 ± 17.5 | <0.0001 |
|  |  | TLC, % pred | 84.0 ± 13.5 | 106.2 ± 17.7 | <0.0001 |
|  |  | DLCO, % pred | 47.9 ± 12.2 | 76.5 ± 10.6 | <0.0001 |
|  |  | CPI | 46.2 ± 11.1 | 21.0 ± 8.1 | <0.0001 |
|  | 6MWD, m | | 299 ± 141 | 480 ± 65 | <0.0001 |
|  | 6MWD <350m | | 11 (61.1) | 1 (2.4) | <0.0001 |
|  | PaO2, mm Hg | | 84.8 ± 10.8 | 92.0 ± 14.6 | 0.043 |
|  | CT score | | 8.0 [25.0] | 7.0 [11.0] | 0.043 |
| Blood | | |  |  |  |
|  | Fibrinogen | | 4.6 ± 1.0 | 3.8 ± 0.8 | 0.002 |
|  | Albumin | | 0.27 ± 0.06 | 0.26 ± 0.04 | 0.444 |
| Quality of life | | |  |  |  |
|  | SF-12 PCS | | 65.2 [12.4] | 61.5 [10.1] | 0.202 |
|  | SF-12 MCS | | 61.6 [27.5] | 68.8 [20.1] | 0.300 |
|  | SGRQ Total | | 38.3 [27.9] | 68.8 [20.1] | 0.009 |
| Sarcopenia assessment | | |  |  |  |
|  | SARC-F | | 3 [2.5] | 0 [0-4] | <0.001 |
|  | RICCI & GAGNON | | 18.7 ± 8.8 | 21.3 ± 7.9 | 0.313 |
|  | FACI-T | | 30 [24.0] | 27 [32.0] | 0.323 |
|  | Grip strenght (women) | | 15 [11.5] | 24 [8.8] | 0.017 |
|  | Grip strenght (men) | | 26 [15.5] | 35 [16.0] | 0.003 |
|  | ALMI (women) | | 6.9 [1.35] | 6.2 [1.0] | 0.246 |
|  | ALMI (men) | | 7.6 [1.5] | 8.3 [1.3] | 0.023 |
|  | Sarcopenia or presarcopenia | | 12 (66.7) | 12 (29.3) | 0.010 |
|  | Body composition | |  |  |  |
|  |  | Fat mass (women) (%) | 45.7 ±1.7 | 43.7 ± 5.1 | 0.412 |
|  |  | Fat mass (men) (%) | 35 [9.7] | 32.6 [6.9] | 0.792 |
| Psychological assessment | | |  |  |  |
|  | HAD Anxiety score^a^ ≥8 | | 5 (27.8) | 4 (10.0) | 0.084 |
|  | HAD Depression score^a^ ≥8 | | 4 (23.5) | 9 (23.1) | 0.971 |
|  | PCLS score^b^ ≥44 | | 5 (33.3) | 4 (10.3) | 0.042 |

Data are expressed as numbers (percentages), mean ± SD, or median [interquartile range]

CPI: Composite Physiologic Index; 6MWD: 6-Minute Walking Distance; PCS: Physical component score; MCS: Mental component score; SGRQ: Saint Georges Respiratory Questionnaire; ALMI: Appendicular lean mass index; HAD: Hospital Anxiety and Depression scale; PCLS: Posttraumatic stress disorder CheckList Scale;

^a^ SP cluster n = 17, NSP cluster n = 39; ^b^ SP cluster n = 15, NSP cluster n = 39.

Table S3. Patient’s demographic characteristics and COVID-19 features at admission in the severe persistent (SP) respiratory impairment cluster and in the non-severe persistent (NSP) respiratory impairment cluster.

|  |  |  | SP cluster | NSP cluster | p-value |
| --- | --- | --- | --- | --- | --- |
| n | | | 18 | 41 |  |
| Male | | | 13 (72.2) | 27 (65.9) | 0.630 |
| Age | | | 67.8 ± 10.9 | 60.2 ± 12.5 | 0.024 |
| Weight loss, kg | | | -6.0 ± 6.0 | -2.8 ± 6.4 | 0.093 |
| Current or ex smoker | | | 10 (55.6) | 18 (43.9) | 0.409 |
| Comorbidities | | |  |  |  |
|  | Obesity | | 8 (50) | 19 (48.7) | 0.931 |
|  | Diabetes | | 7 (38.9) | 7 (17.1) | 0.070 |
|  | Cardiovascular disease | | 13 (72.2) | 22 (53.7) | 0.181 |
|  | Arterial hypertenison | | 12 (66.7) | 18 (43.9) | 0.392 |
|  | Respiratory chronic disease | |  |  |  |
|  |  | COPD | 1 (5.6) | 3 (7.3) | 0.393 |
|  |  | Asthma | 3 (16.7) | 5 (12.2) | 0.640 |
|  |  | Interstitial lung disease | 1 (5.6) | 0 (0.0) | 0.231 |
|  | Inhaled corticosteroids | | 3 (16.7) | 5 (12.2) | 0.453 |
| COVID-19 | | |  |  |  |
|  | Delay between symptoms onset and admission, days | | 5 [9] | 8 [3] | 0.014 |
|  | Symptoms at diagnosis | |  |  |  |
|  |  | Anosmia | 4 (22.2) | 11 (26.8) | 0.708 |
|  |  | Diarrhea | 6 (3.3) | 27 (65.9) | 0.021 |
|  | Total lenght for hospital stay, days | | 46.5 [42.0] | 15.0 [22.0] | 0.075 |
|  | ICU, n | | 10 (55.6) | 26 (63.4) | 0.569 |
|  |  | ICU stay lenght, days | 14.5 [20.0] | 12.0 [10.0] | 0.630 |
|  |  | High flow nasal oxygen, n | 9 (50.0) | 24 (58.5) | 0.822 |
|  |  | High flow nasal oxygen, days | 3.0 [6.0] | 5.5 [5.5] | 0.349 |
|  |  | Intubation, n | 5 (27.8) | 13 (31.7) | 0.915 |
|  |  | Intubation, days | 25.0 [20.0] | 13.0 [19.0] | 0.503 |
|  | Physical medicine and rehabilitation, n | | 10 (55.6) | 12 (29.3) | 0.055 |
|  | Treatment | |  |  |  |
|  |  | Antiviral drugs | 17 (94.4) | 40 (97.6) | 0.542 |
|  |  | Antibiotics | 18 (100) | 41 (100) |  |
|  |  | Corticosteroids | 14 (77.8) | 33 (80.5) | 0.502 |
|  |  | Antithrombotic drugs | 18 (100) | 41 (100) |  |
|  | Complications | |  |  |  |
|  |  | ARDS | 5 (27.8) | 12 (29.3) | 0.965 |
|  |  | Pulmonary embolism | 4 (22.2) | 5 (12.2) | 0.296 |
|  |  | Acute cardiac insuffisciency | 1 (5.6) | 2 (4.9) | 0.891 |
|  |  | Ventilator-associated pneumonia | 3 (16.7) | 5 (12.2) | 0.609 |

Data are expressed as numbers (percentages) or median [interquartile range]

COPD: Chronic Obstructive Pulmonary Disease; ICU: Intensive Care Unit; ARDS: Acute Respiratory Distress Syndrome
